# A Perspective on the Application of Spatially Resolved ARPES for 2D Materials

**DOI:** 10.3390/nano8050284

**Published:** 2018-04-27

**Authors:** Mattia Cattelan, Neil A. Fox

**Affiliations:** 1 School of Chemistry, University of Bristol, Cantocks Close, Bristol BS8 1TS, UK; Neil.Fox@bristol.ac.uk; 2 H. H. Wills Physics Laboratory, University of Bristol, Tyndall Avenue, Bristol BS8 1TL, UK

**Keywords:** spatially localized ARPES, 2D materials, band structure, graphene, transition metal dichalcogenides, 2D heterostructures

## Abstract

In this paper, a perspective on the application of Spatially- and Angle-Resolved PhotoEmission Spectroscopy (ARPES) for the study of two-dimensional (2D) materials is presented. ARPES allows the direct measurement of the electronic band structure of materials generating extremely useful insights into their electronic properties. The possibility to apply this technique to 2D materials is of paramount importance because these ultrathin layers are considered fundamental for future electronic, photonic and spintronic devices. In this review an overview of the technical aspects of spatially localized ARPES is given along with a description of the most advanced setups for laboratory and synchrotron-based equipment. This technique is sensitive to the lateral dimensions of the sample. Therefore, a discussion on the preparation methods of 2D material is presented. Some of the most interesting results obtained by ARPES are reported in three sections including: graphene, transition metal dichalcogenides (TMDCs) and 2D heterostructures. Graphene has played a key role in ARPES studies because it inspired the use of this technique with other 2D materials. TMDCs are presented for their peculiar transport, optical and spin properties. Finally, the section featuring heterostructures highlights a future direction for research into 2D material structures.

## 1. Introduction

The field of two-dimensional (2D) material research began with the discovery of graphene in 2004 [1,2], which seeded the exploration of many new 2D systems. The common features of this group of materials are their extremely small thickness, typically a few atomic layers, with strong in-plane bonds and weak interlayer bonds. Two-dimensional materials are very important in modern technology because of their amazing physical and chemical properties; they are regarded as the thinnest functional materials. 

The family of 2D materials covers the complete range of electrical proprieties from superconducting to insulating [3,4,5,6]. A powerful tool to investigate their properties is Angle-Resolved PhotoEmission Spectroscopy (ARPES). This photoemission technique exploits the emission of photo excited electrons from a crystalline sample by a photon source illuminating the surface. ARPES from conventional light sources allows the probing of the filled states in a material and the direct measurement of its electronic band structure, which is generated by the allowed quantum mechanical wave functions for an electron in a periodic lattice of atoms. Due to the electron momentum sensitivity of the ARPES technique, important characteristics of the materials can be measured such as electron effective mass, Fermi velocity, the Valence Band Maximum (VBM) energy and its position, doping, and many-body effects.

In this review the preparation methods employed to produce 2D material are discussed briefly, and include Chemical Vapor Deposition (CVD) [7], Physical Vapor Deposition (PVD) [8], and mechanical exfoliation. The latter is the easiest way to prepare high-quality 2D materials in a laboratory; it can be applied to the most innovative materials because it is possible as soon as small bulk pieces of a material are produced. The exfoliated flakes typically have micrometric lateral dimensions, but it is difficult to thin down the material to a single layer homogenously. However, the sampling area of a conventional ARPES experiment ranges from tens of micrometers, at synchrotron ARPES facilities, to millimeters in laboratory facilities and can be the principal limitation on the application of ARPES to exfoliated 2D materials.

An ideal experiment to study the band structure of 2D materials should allow the visualization of the sample in the macro or nanoscale, utilizing a small portion of sample material to obtain ARPES. In the past few years, instrument advances have been made that allow complex measurement operations to be performed at synchrotron light facilities and using laboratory-based equipment. In this review, the operating principles of the spatially localized ARPES systems will be presented. First, an overview of early ARPES measurement setups will be discussed before describing state-of-the-art, spatially resolved ARPES equipment. 

This paper features some of the most interesting results obtained to date on graphene, transition metal dichalcogenides (TMDCs), and 2D heterostructures along with discussions about future instrument upgrades for spatially-, spin-, and time-resolved acquisitions. Graphene was a starting point for the investigation by spatially localized ARPES of 2D materials, an example of advanced spatially localized investigations on polycrystalline few layer graphene has been reported [7]. TMDCs have intriguing electronic, spintronic, and photonic properties, and the study of their band structure is of fundamental importance. Examples of the most well-known TMDC, namely, MoS_2_ [9], and potentially the most innovative such as TiSe_2_, VSe_2_ [10], and ReS_2_ [11,12] are also mentioned. In the section on 2D heterostructures several examples of graphene/TMDC composites are presented. Graphene is often used as an active component of the heterostructure [13] but cases where graphene is employed as protective capping layer [14,15] as well as a conductive substrate [11,15] have also been reported. A rare example of a study on an "all-TMDCs’ heterostructure is presented in this section [15] exemplifying the strength of this technique for the study innovative ultra-thin 2D devices. 

## 2. ARPES Setups: Technical Considerations 

### 2.1. ARPES Technical Considerations

The ARPES technique needs an instrument setup that allows (i) the possibility to investigate the photoemission intensity as function of emission angle and (ii) the ability to distinguish the kinetic energy of the photoelectrons. A sample for ARPES must be crystalline to possess an ordered band structure, and its surface must be smooth to conserve the k-parallel component from the crystal to the vacuum [16]. The surface must also be ultra-clean because the sampling depth is typically only few nanometers. ARPES is therefore performed in ultra-high vacuum (UHV) chambers to analyze a clean surface and to ensure that gas molecules do not scatter the low energy photoelectrons. 

**Figure 1:**
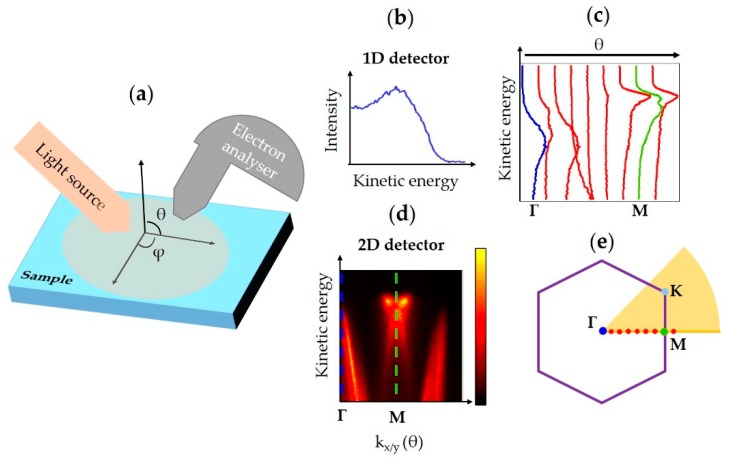
(**a**) Conventional Angle-Resolved PhotoEmission Spectroscopy (ARPES) scheme, θ and φ represent the polar and azimuthal angles respectively; (**b**) 1D detector spectrum in Γ, blue point in (**e**). Using 1D detectors spectra must be acquired for every θ and φ; (**c**) A series of 1D acquisitions in a polar scan, in which the blue and green spectra represent data acquired for the material at Γ and M points respectively. The location of the spectra in the 1st Brillouin zone of the example material (TiSe_2_) is represented by the dots in (**e**); (**d**) Example of a 2D detector acquisition. The blue and green dashed lines mark Γ and M points respectively. The acquisition plane is represented by the yellow line in (**e**); (**e**) Representation of the 1st Brillouin zone of the example material, three of the high symmetry points Γ, M and K are marked. The purple solid line is the 1st Brillouin zone of the material, and the shaded orange area sketches the acquisition sector of a 2D analyzer with deflection mode.

In the very first ARPES experiments, the samples were a few millimeters wide and photoelectrons were detected using a hemispherical analyzer with a small acceptance angle and a simple electron counting detector. The role of the electron analyzer is very important; indeed, it is the element that allows one to measure the photoelectron kinetic energy, creating, with the simplest 1D detector, an intensity versus kinetic energy spectrum (Figure 1b). In the early experiments, electron analyzers were also used to select a small solid angle of the whole photoelectron emission cloud, which corresponded to a small portion of the k-space [16]. Samples were illuminated with synchrotron light or discharge lamps over a relatively large and homogenous area, see Figure 1a. When the analyzer was positioned at the sample normal, the photoelectrons emitted from it came from the Γ point i.e., the center of the Brillouin zone, see Figure 1b,e. To perform ARPES the sample had to be rotated in both polar and azimuthal angles so that the electron analyzer acquired photoemission spectra at different angles (Figure 1c). By combining the photoemission signal acquired at different angles a reconstruction of the band structure of the material was obtained.

With 1D detector mapping the acquisition of a full band structure was time-consuming and needed complex sample movements. The introduction of 2D electron detectors provided the means to reduce the number of sample rotations, as illustrated in Figure 1d. Electron analyzers equipped with multichannel plate 2D detectors can discriminate the electron dispersion in the analyzer plane allowing the acquisition of a slice of the k-space, as shown in Figure 1d, e, which can be equivalent to a polar scan with a 1D detector (Figure 1c). This technological improvement drastically increased the quality of spectra obtained and decreased the acquisition times for conventional ARPES. The significance of this advance can be seen by comparing Figure 1c,d.

A recent enhancement to these analyzer systems has enabled angular scans to be made in two dimensions in k-space without tilting or rotating the sample. The scanning of k-space is carried out electronically using dedicated deflectors. An example representation of the region of k-space that can be sampled with these detectors is represented by the orange shaded area in Figure 1e. Consequently, using such electron analyzers, a sector of k-space can be acquired. Using a suitable photon source energy, it is possible to acquire a representative set of high symmetry points without any mechanical movement of the sample. Keeping the sample in the same position during k-space mapping is a crucial requirement for obtaining spatially localized ARPES.

### 2.2. Spatially Localized ARPES

Considering the complex procedure needed to perform conventional ARPES from large area samples, it is understandable that to perform ARPES on micro- or nano-sized samples poses a significant technical challenge. Instruments need to scan a vast region of the sample surface in real space (X/Y plane) to find the interesting regions, typically microns wide, and then perform ARPES on them. Nowadays there are different setups available to research and industry to be able to do spatially localized ARPES, and the instrument configurations can be divided into the following two categories:

1. setups that present extremely small light spots, and where surface mapping is done by moving the sample with respect to the light;
2. setups that allow visualization of the real and k-space by extracting electrons using strong electric fields.

**Figure 2:**
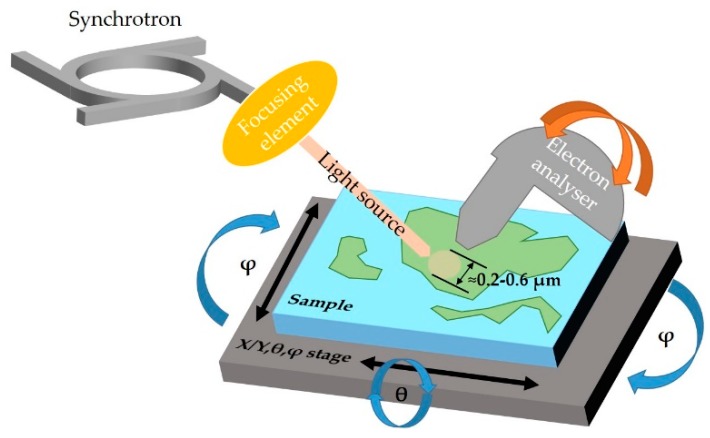
Schematic representation of spatially localized ARPES with synchrotron light source setup. The radiation from the synchrotron is focused on a spot of few hundreds of nanometers. The sample can be visualized by scanning the real space (X/Y) and collecting photoelectrons with the energy analyzer. To perform the angle resolved acquisition either the analyzer can move as represented by the orange arrows (Spectromicroscopy setup) or the sample can rotate (blue arrows, ANTARES, I05, MAESTRO setups). The electron analyzers are equipped with 2D detectors (see Figure 1d) and therefore they can analyze a part of the reciprocal space (see yellow line in Figure 1e) without moving the sample. If equipped with deflectors, as in ANTARES, it becomes possible to visualize a sector of the k-space without moving the sample.

For the first category, the spatially-localized ARPES is typically carried out at synchrotron light source facilities, see Figure 2. They differ from classical ARPES beamlines in that the focusing of the beam is done by dedicated focusing elements. Synchrotron light must have a high brightness and photon flux to offset the strong attenuation by the focusing elements.

With these setups it is possible to visualize samples in real space by mounting them on motorized stages (Figure 2) and collecting photoelectrons as function of the sample position. The lateral resolution is directly linked to the beam spot size: the smaller the spot, the better the lateral resolution. 

ARPES mapping is acquired either by moving the electron analyzer in UHV over a range of different emission angles, which is the configuration used at the Spectromicroscopy beamline in the Elettra synchrotron [17], or, most commonly, by rotating the sample with respect to a fixed analyzer, as shown by the arrows in Figure 2. The latter configuration introduces the non-trivial issue of keeping the photon beam in the same sample position while rotating the sample but allows the use of a large hemispherical electron analyzers that are not movable under UHV conditions. This setup is employed at the beamlines I05 in Diamond, ANTARES in Soleil [18] and MAESTRO in Advanced Light Source (ALS). All of these facilities refer to spatially localized ARPES as “nano-ARPES” because the light spot can be focused to nanometer-sized spots [19]. The use of state-of-the-art hemispherical electron analyzers with a deflection mode, as in ANTARES, allows the sample to be kept in a fixed position and the measurement of a larger sector of k-space can then be acquired, as seen in Figure 1e.

The synchrotron light source is important not only for the brilliance of the light, but also because it can allow ARPES to be performed at different photon energies and polarizations. Moreover, the broad range of photon energies available allows X-ray photoemission spectroscopy (XPS) to be performed. The ability to perform spatially localized core-level spectroscopy gives a much deeper insight into surface conditions such as composition, oxidation and contamination.

**Figure 3:**
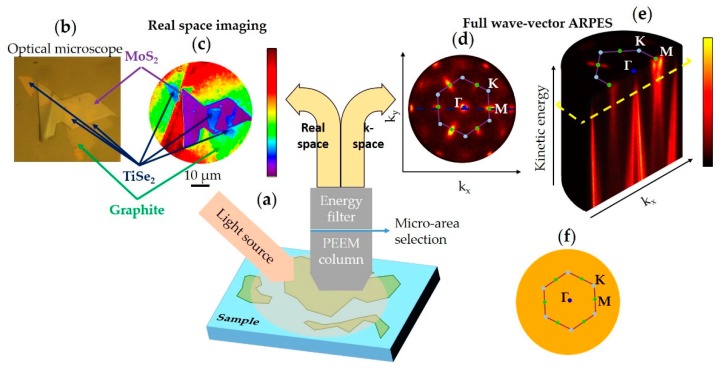
(**a**) Schematic representation of spatially-localized ARPES acquired by an energy-filtered PhotoEmission Electron Microscope (EF-PEEM); (**b**,**c**) Real space sample visualization of a 2D heterostructure; (**b**) Optical microscope image; (**c**) Real space image obtained with a single snapshot of 20 s acquired close to the work function threshold. The areas of interest can be selected by micro-apertures in the image plane; (**b**,**c**) Blue, purple and green arrows represent TiSe_2_, MoS_2_ and graphite flakes respectively; (**d**) Single snapshot of full-wavevector slice of 60 s of TiSe_2_ acquired from one single flake of about 20 microns. The kinetic energy of the image (**d**) corresponds to the dashed yellow plane in (**e**); (**e**) Series of snapshots at different kinetic energies to form a complete ARPES map, the data cube has been cut along the dashed blue line in (**d**). The cut data shows the classical ARPES acquisition, i.e., kinetic energy vs. electron momentum; (**f**) Representation of the 1st Brillouin zone of the example material, in orange circular the field of view of the full-wavevector ARPES in (**d**,**e**). (**d**–**f**) Three of the high symmetry points are marked with Γ, M and K; the blue dot, the green dots and light blue represent the Γ, M and K point respectively. The purple solid line represents the 1st Brillouin zone. The real and reciprocal space images reported in this figure were acquired at Bristol NanoESCA facility and have not been published previously.

For the second category, the photoelectrons are accelerated by an extractor electrode towards an electron optical column, which contains electrostatic or magnetic electron lenses, corrector elements such as stigmators and deflectors, apertures placed in the image plane and contrast apertures. Such instruments are usually referred to as PhotoEmission Electron Microscopes (PEEMs), see in Figure 3a. These setups can be configured to visualize how the photoelectrons escape from the sample at different angles (in k-space) (Figure 3d,e) and by imaging the sample in real space allow the identification of features of interest (Figure 3b,c). Several field of views are available both for real and reciprocal space operation. Apertures positioned in the image plane are used to select a micron-sized portion of the sample whereas contrast apertures select specific emission angles. 

These instruments allow the sample to be imaged without moving it either in real or reciprocal space (Figure 3c,d). This important feature solves major alignment, rotational and movement problems under UHV conditions. Another advantage of these setups is that the zones selected for micro-ARPES as well as the lateral resolution are not dependent on the light spot size, therefore laboratory light sources such as discharge lamps can be used, making it feasible to have PEEM on UHV laboratory-based platforms.

However, a PEEM column cannot filter the photoelectron kinetic energy, therefore it is missing an ARPES measurement requirement. To distinguish the kinetic energy of photoelectrons, PEEMs must be equipped with energy filters that allow one to scan the photoelectron kinetic energy spectrum, these more complex setups are called energy-filtered PEEM (EF-PEEM). The ARPES mapping operation is acquired by imaging at a range of kinetic energies of the full-wavevector landscape (Figure 3d), allowing direct imaging of isoenergetic slices of the band structure (see Figure 3d and yellow dashed plane in Figure 3e). The full 1st Brillouin zone can be acquired without moving the sample in micron-sized areas, see Figure 3d–f. It should be noted that the isoenergetic images, both in the real and reciprocal space, are acquired with no electronic and/or mechanical scanning, but by capturing a single snapshot of the complete field of view presented by the instrument; this ability enables very fast ARPES acquisitions.

Included among the available commercial EF-PEEM instruments are the NanoESCA II and the Time-of-flight (TOF) PEEM of Focus (Focus GmbH, Huenstetten, Germany), the METIS and FE-LEEM (low energy electron microscopy)/PEEM P90 of SPECS (SPECS GmbH, Berlin, Germany), and the ELMITEC PEEM/LEEM (ELMITEC Elektronenmikroskopie GmbH, Clausthal-Zellerfeld, Germany). The energy selection in these instruments is performed in a range of different ways, including using hemispherical analyzer(s), TOF filters and electrostatic/magnetic retarding optics.

The best energy resolutions are obtained by the hemispherical analyzers and TOF, for example NanoESCA II is equipped with two hemispherical analyzers coupled in a “S” fashion to minimize the aberrations [20], and with these setups the achievable energy resolution is typically of the order of few tens of meV. The PEEM/LEEM instruments do not excel in energy resolution, however, they are very versatile instruments and with their electron source they can perform low energy electron diffraction (LEED) in micro-spot mode and visualize single atomic steps [21].

All these instruments have a lateral resolution in the nanometer range, LEEMs in this respect typically out-perform PEEMs, but in any case, spatially localized ARPES is usually performed over an area of a few microns and limited by the fact that the area is selected by means of a mechanical aperture.

Laboratory-based equipment typically use discharge lamps, such as a He lamp that offers 21.2 eV (He I) or 40.8 eV (He II) photon energies. The poor photon tunability does not represent a problem for single layer 2D materials because they do not have a k_z_ dispersion, but it is a limitation for 3D materials where synchrotron tunable radiation must be employed to analyze the k_z_ dispersion. Indeed, several EF-PEEM are installed on synchrotron light source facilities to offer the combination of high flux and versatility of synchrotron light with the fast imaging and stability of the PEEM. Almost all the synchrotron light facilities host a PEEM beamline, among those facilities there are NanoESCA [22] and Nanospectroscopy beamlines in operation at Elettra, I06 at Diamond, HERMES at Soleil, and SIM at PSP.

It is important to note that using a technique called dark-field PEEM a portion in k-space and energy can be selected [23]; this defined element in k-space, e.g., a Dirac cone, can be used as source of signal to image the sample in real space, allowing the identification of the material portion where the k-space feature arises. These acquisitions can be performed with EF-PEEMs [23] and in some nano/micro-ARPES beamlines such as Spectromicroscopy [24] and ANTARES [18].

### 2.3. ARPES Setups Comparison

The next section is principally focused on spatially localized band structure measurements nonetheless other types of ARPES setups are included in the context of some of the most representative 2D materials. Each type of ARPES setup presents its own pros and cons which are reported in Table 1 along with future technique upgrades.

## 3. Spatially Resolved ARPES for 2D Materials

Spatially localized ARPES has been developed to allow the analysis of micro- or nanometric samples, consequently it is strongly dependent upon the lateral dimensions of the sample. Several technological challenges arise from the production large flakes of 2D materials, therefore it is important to understand how they can be synthesized. Three different techniques to grow 2D materials have been reported:

1. **Epitaxial growth** by CVD or PVD. This method allows flat and azimuthally oriented layers to be deposited on large single crystal substrates. It is easy to control the number of layers by changing the deposition time, growth chambers can be directly mounted onto ARPES equipment so that samples can be transferred for analysis under UHV conditions. With epitaxial films it is not necessary to employ spatially-resolved ARPES to limit the analyses region on single material grains because the grains are azimuthally oriented with the substrate and form a macroscopic continuous ordered crystalline lattice. Because of the large amount of signal available for ARPES, advanced analyses such as spin-resolved or time-resolved ARPES are feasible on such samples (see in Table 1). There are two main disadvantages of this method: it is time consuming, because evaporators and setups must be carefully optimized for each different material; secondly, the interaction between the substrate and 2D materials is so strong that usually it is not feasible to transfer the 2D layers onto other substrates.
2. **Conventional CVD** produces large micron-scale grains azimuthally misaligned. In respect to the epitaxial growth, the control of the number of layers it is more difficult and occasionally multi-layers are produced. Furthermore, this method is more prone to contamination because it is usually performed under non-UHV conditions. The advantage of conventional CVD with respect to epitaxial growth is that it is possible to use single crystal and polycrystalline substrates with weaker bonds to the 2D material, allowing their detachment and transfer.
3. **Mechanical exfoliation**, also called “adhesive tape technique”, produces extremely high-quality layers because they are peeled from ultra-pure single crystals. The main drawbacks are the micron-sized areas of the layers and that the thickness is not easily controllable. This technique is the main method used for studying promising new 2D materials because it is fast and easily achievable, exploiting the ease of exfoliation along the weak van der Waals inter-layer bonds. Importantly, it is the main technique to form 2D heterostructures.

Not all the materials can be prepared using all three methods. Some analogues of graphene such as silicene [25] and germanene [26], and some TMDCs, such as PtSe_2_ [27], are obtainable with good quality only by epitaxial methods. Indeed, spatially localized ARPES is ideally suited to the analysis of samples prepared by CVD and mechanical exfoliation. Arguably the most interesting samples for study are the mechanically exfoliated ones, due to their formation in micrometric flakes with multiple layers. These can be used to analyze the changes in band structure and electronic properties with different numbers of layers on a single sample. For the most challenging 2D material analyses, spatially localized ARPES is also used to analyze bulk crystals, which, at the beginning of the material growth optimization, present only a few microns of clean terraces. The typical procedure followed for the analysis of a new 2D materials would be:

1. characterization of the bulk material band structure;
2. analysis of 2D exfoliated layers to observe quantum confinements effects and any difference with respect to the bulk;
3. finding a method to produce large 2D layers and perform advanced characterizations such as spin-resolved, or time-resolved ARPES studies.

Some examples of spatially localized ARPES studies on graphene, TMDCs, and 2D heterostructures are presented below which show how insightful this technique can be for exploring the 2D materials world. 

### 3.1. Graphene and Its Analogues 

The starting point of ARPES investigations on 2D materials has been graphene, and the development of spatially localized ARPES setups has to some extent been driven by graphene [2,28] and more recently by other 2D materials [3]. Nowadays graphene and graphite are so well known that they are used as calibration samples in ARPES facilities [18]. 

**Figure 4:**
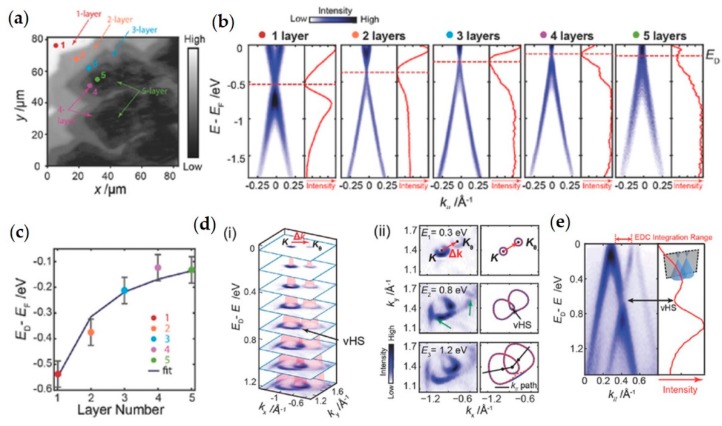
(**a**) Large-scale spatially scanned image of the graphene and few layer graphene on copper foil obtained by acquiring photoelectrons in the spectral range of the copper *d*-bands. Dots with different color mark selected positions for subsequent measurement; (**b**) Energy-momentum dispersion taken at positions shown in (**a**). Red dashed lines indicate the energy of the Dirac point (E_D_) in each spectrum of the top layer. Red solid curves are integrated energy distribution curves (EDCs) for each spectrum, which are corrected by the Fermi–Dirac distribution, allowing one to see features near the Fermi surface; (**c**) Evolution of the value of E_D_ measured from the top layer with total number of layers. The blue line indicates a fit of the data using the capacitor-model; (**d**) (i) Equal energy contours of twisted bi-layer graphene band structure with twist angle 8.2° showing two Dirac cones, (ii) Comparison between measured and predicted energy contours. The dotted red curves are calculated from a tight binding model for the overlapping bands from two Dirac cones without hybridization. The solid blue curves show a guide to the eye to illustrate the hybridization effect. The mini gaps marked by green arrows stem from a Moiré super-potential; (**e**) Energy-momentum dispersion passing through the two Dirac points and the vHS. The right panel shows the integrated EDC over the region shown above. Adapted from Ref. [7] with permission of ^©^2017 WILEY-VCH Verlag GmbH & Co. KGaA, Weinheim, Germany.

Graphene is a single layer of sp^2^ hybridized carbon atoms. This material is considered a semimetal, the electrons and holes act like Dirac fermions with the points of intersection between the conduction and valence bands being called Dirac points. The energy versus k dispersion curves of electron and holes form two-dimensional cones around the Dirac points (see Figure 4b), which are usually referred to as “Dirac cones”; for a charge neutral layer, the “Dirac point” is at the Fermi level. There are six points inside the first Brillouin zone where the conduction and the valence bands meet.

ARPES can image directly the Dirac cones and electronic information can be extracted such as doping [29], i.e., the difference between Fermi energy and Dirac energy, Fermi velocity [30] and many body interaction, such as electron-plasmon and electron-phonon coupling [31]. 

The atomically thin nature of graphene makes its electronic properties strongly influenced by the substrate and any surrounding ultra-thin layers. Graphene has been extensively studied by conventional ARPES because it is relatively easy to obtain epitaxial layers by dosing carbon precursors on hot catalyst single crystal substrates, such as Ni(111) [32,33] and Ru(0001) [34]. Its band structure has been comprehensively investigated as a function of the metal substrate by ARPES. It has been found that some metals interact so strongly with graphene that they cause a drastic change in its band structure [32,35,36,37], while others are considered weakly interacting and graphene placed on them is considered quasi-freestanding [32,33,38]. To change the graphene/substrate interaction a methodology, called intercalation, has been developed to allow the exchange of the graphene support. In these types of experiments, graphene is exposed to an agent that can intercalate it, i.e., it positions itself in between graphene and substrate. Another interesting method to change the properties of graphene is by surface doping using alkaline metals deposited on top of it [39,40]. This doping method is also used for 2D semiconducting layers to visualize their conduction bands as reported later in this review. 

The properties of graphene in contact with different species such as substrates, intercalating agents, and deposited species, can be studied by ARPES. For example graphene intercalated with weakly interactive metals or oxygen can remove hybridization with the substrate [32,36,38,41], or strongly interactive metals can deliberately induce hybridization to tune the material properties [42,43]. For example, graphene spin degeneracy can be lifted by intercalation of one mono-layer of ferromagnetic metals [42], or a particular spin structure can be obtained intercalating 1 mono-layer of low interactive metal underneath graphene grown on a ferromagnetic substrate [36]. Spin sensitive detectors for ARPES are essential to understand the graphene spin structure [36] and, as reported later, they are even more important for some semiconducting TMDCs for their intrinsic spin-splitting band structures.

The studies on spatially localized ARPES on graphene have been fundamental for the investigation of azimuthally disordered CVD-grown graphene. This analysis has been carried out with graphene on copper [7,44,45,46,47,48,49,50,51,52], on platinum, [42,53,54], on silicon carbide [55,56], or to study multi-layer regions that do not cover all the surface, such the one found on copper [7] and ruthenium [57,58].

One important study that emphasizes the power of spatially-localized ARPES for graphene was carried out with multi-layer graphene on copper [7]; some of the key results of this work are reported in Figure 4. In this study [7] micro-ARPES has been carried out on graphene and twisted multi-layers of graphene. The aim of the paper was to study the evolution of the doping caused by the substrate (Figure 4a–c) and to study the interaction between the layers (Figure 4d,e). In respect to doping, it has been found that with increasing numbers of layers, the top layer of graphene becomes less electron-doped, i.e., the Fermi energy it is closer to the Dirac energy (see in Figure 4b), which can be explained by an effective capacitor model of the multilayer system. Due to the existence of an effective work function difference, the electrons will transfer from the copper substrate to the graphene, filling the unoccupied states, which causes the doping effect (see Figure 4b). As the number of layers increases, the top layers are shielded from the substrate, and accumulate fewer transferred electrons than the lower layers, see in Figure 4b,c. The interest in studying the interaction between the layers, is related to the way in which the twisted multilayers interact each other and form van Hove Singularities (vHS) that are detectable by mapping the band structure of bi-layers and multi-layers twisted at angles of up to 31°. Figure 4d and e show the vHS analysis for a bi-layer twisted by 8.2°, the band structure of the two layers clearly interacts, there is a decrease of the photoemission intensity where the two Dirac cones intersect, as seen in Figure 4e. In both aspects performing ARPES in a micron-sized region (see Figure 4a) of the sample has been essential for visualizing the doping trends and the vHS versus angle measurements.

The example study reported in Figure 4 [7] also demonstrated that it is very important to map a relatively big portion of the k-space (see Figure 3d–f) to perform cuts, i.e., energy versus k_//_, in all the desired directions. For example, the spectra in Figure 4b are acquired in the perpendicular direction of the Γ→K direction whereas in Figure 4e the cut is along the two Dirac points of the twisted layers. The ability to map a sector of the k-space also allows one to obtain isoenergetic maps as shown in Figure 3d and Figure 4d. In this respect instruments that can perform full-wavevector ARPES, such as the EF-PEEM, will probably be favored for such complex acquisitions in the future.

Important spatially localized ARPES studies have been acquired on mechanically exfoliated graphene flakes [59], on bi-layer graphene to study the vHS [45,60] and on 2D heterostructures, the latter are reported later in this review. Because spatially-localized ARPES setups can also visualize the material in the real space (see discussion in Section 2.2 and Figure 4a) studies of reaction processes involving graphene, such as oxidation or intercalation, can be carried out in real time, especially using PEEM (see discussion in Section 2.2). ARPES analysis can link the material transformation and chemical reaction with changes in electronic properties [44,61,62]. 

All these studies that focused on graphene demonstrate a methodology that can be applied to the study of other 2D materials, confirming the pivotal role that graphene had as the prototype 2D material. 

Hexagonal boron nitride (*h*-BN), the analogue of graphene with boron and nitrogen atoms, has been synthesized and studied in a similar fashion to graphene. It is an insulator with a band gap of more than 5 eV [63] and therefore represents a fundamental component of future 2D electronic devices. Hexagonal boron nitride single layers have been grown on epitaxial substrates and intercalated to obtain a quasi-free-standing layer and this change has been visualized by conventional ARPES [64,65]. It has also been mechanically exfoliated and analyzed by nano-ARPES [66], exactly as has been done previously with graphene. 

Another interesting analogue of graphene is phosphorene, i.e., a single layer of black phosphorous. It has a notable technological advantage when compared with graphene which is a direct and sizeable band gap for a mono-layer up to bulk films in the eV range, making it very interesting for optoelectronic devices [67]. Phosphorene is obtainable by mechanical exfoliation of black phosphorus but interestingly only the bulk material has been extensively studied by ARPES [68,69,70,71,72,73,74] whereas the mono-layer has not been investigated yet, probably because it is prone to air oxidation [75]. As reported later in the heterostructure Section 3.3, advanced techniques for the protection by encapsulation with oxidation resistant materials, such as graphene and *h*-BN [14], will probably be applied to obtain single layer phosphorene band structure by spatially-localized ARPES.

### 3.2. Transition Metal Dichalcogenides

TMDCs are a very important class of 2D materials. They comprise three atom layers, a central layer composed of a transition metal and an upper and lower layer made up of chalcogenide atoms, typically sulfur, selenium, and tellurium [76]. In contrast to graphene, an individual TMDC mono-layer can have different phases, the most typical for a mono-layer are the so-called 2H and 1T, which have a AbA and AbC stacking of lattice atoms, respectively (with the capital and lower case letters denoting chalcogen and metal atoms, respectively) [76].

So far, group VIB TMDCs have kindled the greatest amount of interest among researchers in the electronic, optoelectronic and spintronic fields [77]. Several TMDCs of this group are semiconductors with a band gap in the eV range, and they can therefore be efficiently integrated into ultra-thin field-effect transistors (FET) devices. This has been the prime motivation for researching them. Their intrinsic semiconductor characteristic is responsible for promoting a good on/off ratio in FET devices easily outperforming graphene with its semi-metallic band structure. Molybdenum and tungsten chalcogenides are naturally layered materials with a 2H stacking. Their band structure varies as a function of the number of layers. In their bulk form, most of these TMDCs present an indirect-gap characterized by a VBM at the Γ point and a Conduction Band Minimum (CBM) at the midpoint Σ along the Γ-Κ high symmetry directions. When the thickness is reduced to a single mono-layer, the band gap becomes direct at the Κ-points.

**Figure 5:**
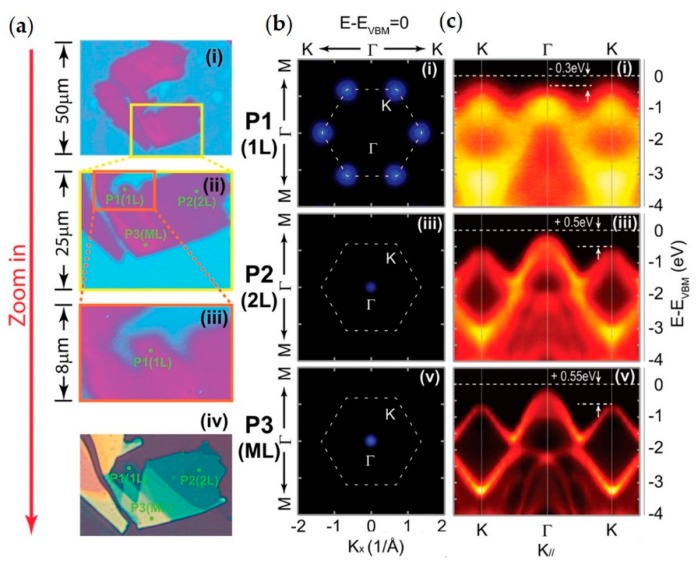
Band valley evolution from multi-, bi- to mono-layer MoS_2_ nanoflakes. (**a**) 2D photoemission spectra intensity contrast map of MoS_2_ flakes (measured at the Fermi level), with different magnifications from large area (i) to small area (iii), showing the procedure to locate the targeted mono-layer flake. Panel (iv) gives the optical image of the same flake, where the mono-, bi- and multi-layer MoS_2_ flakes can be clearly seen. Points P1−P3 indicate the three measurement positions for mono-, bi- and multi-layer MoS_2_ flakes; (**b**) Constant energy plots measured at mono-layer (point P1), bi-layer (point P2), and multi-layer (point P3) regions, with the energy positions at E − E_VBM_ = 0 eV; (**c**) Band dispersions along the high symmetry K-Γ-K direction from points P1−P3, showing the band valley evolution with different flake thicknesses. Adapted with permission from Ref. [9]. Copyright 2016 American Chemical Society.

The modification of the band structure as a function of the number of layers for MoS_2_ has been demonstrated by micro-ARPES [9,78], this transformation arises from quantum confinement effects. In Figure 5 an example of micro-ARPES on MoS_2_ exfoliated flakes is reported. In Figure 5a optical and PEEM images of the sample are presented, while in Figure 5b,c VBM and ARPES along Γ-K direction are shown, respectively. The switching of the VBM from mono-layer to thicker layers is clearly detectable when comparing the results in Figure 5b. With this experiment of spatially resolved ARPES on only one deposited sample has proved possible to show that the modification of the band structure of ultra-thin TMDCs layers occurs, proving once again the strength of this technique combined with mechanical transfer method. 

One fascinating feature of this class of semiconducting TMDCs is their band structure around the K points which is composed by two spin-polarized branches due to the strong spin-orbit interaction originating from the transition metal ion’s *d* orbitals [79]. For example, for mono-layer WSe_2_ the VBM is characterized by two bands with a spin-splitting of about 0.5 eV, making this material one of the most studied for spintronic devices [80,81]. The lack of inversion symmetry in mono-layer TMDCs creates unequal K and K′ valleys, the spin projection along their out-of-plane axis is well defined, and the two split bands are spin-up and spin-down. The time-reversal symmetry requires that the spin splitting must be opposite at the two distinct valleys, leading to a spin-valley locking relationship [82]. Electrical generation and control of the valley population has been achieved [83] and it is the crucial point of the emerging field called “valleytronics”. The prospect of valley manipulation offers a new vehicle for the transfer of information to augment those provided by charge and spin [84].

For this class of materials, the implementation of a spin-resolved detector for ARPES is of crucial importance. Until now the spin-resolved ARPES studies have been conducted on bulk TMDCs [85,86,87,88] and on epitaxially-deposited TMDCs [80]. These studies are rare and challenging because they need high brightness and long acquisition times. Consequently, the reduced light intensity experienced with micro/nano ARPES makes these acquisitions very challenging (see Table 1). However, a recent development in PEEM at the NanoESCA beamline in Elettra which has been equipped with a spin-detector [89] will probably solve this problem allowing the full-wavevector spin-resolved ARPES measurements to be made on small exfoliated flakes [90].

Because several TMDCs are semiconductors it is of pivotal interested to study their conduction band, and this may be accomplished by two different approaches: (i) deposition of alkaline metals followed by ARPES and (ii) time resolved pump and probe ARPES.

As stated above for graphene, doping the surface by deposition of alkaline metals it is a well-known method to shift the Fermi energy of the material and, in the case of semiconducting TMDCs, allows analysis of the CBM [8,9,91,92,93]. The use of alkali metal deposition can also cause modification of the band structure of the material because it creates an electric field perpendicular to the sample surface. It has been found that this field can induce a Stark effect [92,94,95] and change from an indirect to direct band gap in bulk MoSe_2_ [92]. Another interesting feature is the formation of a two-Dimensional Electron Gas (2DEG) on the sample surface after the alkali metal deposition, opening new opportunities for advanced electronic and quantum devices [92,94]. In the literature, examples of spatially resolved ARPES where the sample has been doped with alkaline metals [96] are rarely presented probably because of the intrinsic complexity of depositing metal during the ARPES acquisitions. For instance, in EF-PEEM instruments there is only a limited space, typically a few millimeters, between the sample and the extractor lens.

Another way to study the conduction band of the sample is time-resolved ARPES involving “pump and probe” experiments. In these experiments, two photons with a femtosecond delay are sent to the sample. A photon source called “pump” has the function to excite electrons into the conduction band, while the other source called “probe” has the role of extracting photoelectrons. This technique is used to reveal the dynamics of the charge carriers and it is the only method that is able to visualize the electron relaxation path in excited states with momentum resolution [97]. Similar to spin-resolved ARPES, time-resolved ARPES has been carried out on bulk crystals [97] or epitaxially-grown MoS_2_ [98,99], because their large area overcomes the usual problems of low signal and the long acquisition times (see Table 1). Future enhancements to this type of technique include the employment of time-resolved sources for EF-PEEM [100] and TOF PEEM [101,102]. It is envisaged that the latter will be crucial in the future to perform spatially- and time-resolved ARPES.

Other TMDCs, such as TiSe_2_ and VSe_2_, are metallic. They have a 1T stacking and show low temperature transitions to states with charge density waves (CDW) leading to periodic modulations of the electronic charge density. The resulting superlattices can be either commensurate or incommensurate and the CDW ordering can compete with other phenomena such as superconductivity and anti-ferromagnetism. ARPES investigation of these materials is still at an early stage, these materials have been only analyzed by ARPES in their bulk form [103,104] or as epitaxial mono-layers [103,105].

**Figure 6:**
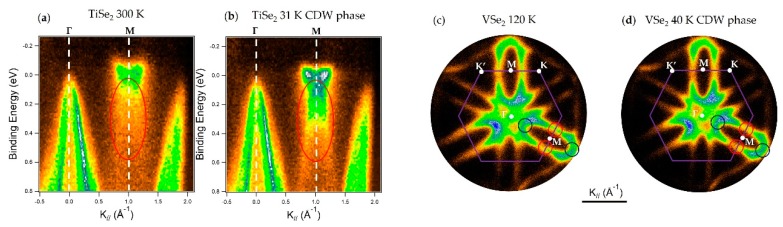
Example of TMDCs with CDWs. (**a**,**b**) TiSe_2_ bulk ARPES acquisition along Γ→M direction at (**a**) 300 K and (**b**) 31 K. The CDW folding of the band structure is detectable for the replica at low temperature for the Se 4*p* features at the Γ point, red circles zones. The Γ and M points are indicated by dashed white lines; (**c**,**d**) VeS_2_ bulk ARPES Fermi surface snapshot at (**c**) 120 K and (**d**) 40 K. The CDWs Fermi surface gapped and un-gapped regions are indicated by the red and blue circles, respectively. The purple solid line represents the 1st Brillouin zone of the material, the white dots and letters indicate the high symmetry points. All these images were acquired at the Bristol NanoESCA facility and have not been published previously.

Figure 6 shows micro-ARPES acquisitions on TiSe_2_ and VSe_2_ small bulk crystals. The CDW effects in TiSe_2_ are visible in the acquisitions along the Γ→M direction; the replica of the photoemission features in Γ, due to the Se 4*p* orbitals, is visible in Figure 6b at the M point (red circle). The replica is due to the formation of the (2 × 2) CDW phase [103]. The band structure modifications due to the CDWs in VSe_2_ are much more subtle and they are still under investigation [10]. Their visualization requires snapshots of the Fermi surface as illustrated in Figure 6c,d, which show well-defined “pockets” surrounding the M points. By acquiring Fermi surface snapshots at different temperatures in the CDW phase, a small gap opening (few meV) is observable which induces a reduction in intensity along the K’-M-K direction, shown as red circles in Figure 6c,d, whereas other parts of the “pockets” do not decrease in intensity at the same rate (blue circles). It is important to note that these latter measurements are extremely complicated to acquire with conventional 2D detectors because several azimuthal rotations are needed to investigate a whole “pocket”. The use of full-wavevector ARPES notably improved the acquisition times for such complicate experiments.

**Figure 7:**
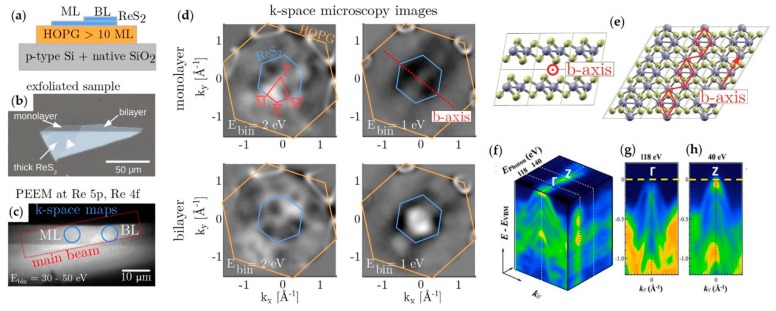
ReS_2_ spatially resolved ARPES acquisitions. (**a**) Sketch of the exfoliated few-layer ReS_2_ sample; (**b**) Optical microscope image of the sample before transfer onto HOPG; (**c**) Real space PEEM image with E_binding_ integrated over 30–50 eV (Re 5*p*/Re 4*f* core levels); (**d**) Second derivative k-space images selectively measured on the mono-layer and bi-layer areas of the sample with the surface BZ of ReS_2_ indicated in blue and of HOPG in orange. The high symmetry directions are marked by red lines; (**e**) Distorted 1T crystal structure of ReS_2_ with the Re chains forming along the *b*-axis of the crystal indicated in red. Adapted with permission from Ref. [11]. Copyright 2017 American Chemical Society. Nano-ARPES acquisition of bulk ReS_2_ (**f**) nano-ARPES signal (blue = low to orange = high) as a function of energy below the Fermi energy (vertical axis) and in-plane momentum k_//_, for excitation energies of 118 and 140 eV (left and right respectively); (**g**,**h**) panels show the nano-ARPES electronic dispersion of the valence bands at the Γ and Z points of the 3D Brillouin unit cell. Adapted with permission from Ref. [12]. Copyright 2017 Springer Nature.

A recently studied TMDC is ReS_2_ for its unusual in-plane anisotropy. The ReS_2_ structure is considered a distorted 1T crystal structure and when compared to the 2H structure of group VIB TMDCs, an additional valence electron leads to the formation of Re chains along the b-axis of the crystal, see scheme in Figure 7e. The low crystal symmetry results in highly anisotropic optical, vibrational, and electron transport properties and therefore adds an additional degree of interest for applications in sensor and electronic devices. This material is at a very early stage of its study, and therefore studies of spatially localized ARPES are pivotal not only for probing layers with different thickness [11] but also for investigating small bulk pieces [12,106]. Figure 7a,b features the first spatially localized ARPES on ReS_2_ single and bi-layers. While Figure 7f–h show the ARPES from a small bulk crystal using different photon energies to explore the k_z_ dispersion. From the full-wavevector ARPES reported in Figure 7b the predicted lack of hexagonal symmetry of this material is easily detectable. The unusual symmetry has been demonstrated by acquiring a single snapshot exemplifying once again the importance of the full-wavevector ARPES. Interestingly, the direct/indirect band gap transition of the ReS_2_ has not been clearly established and further studies will have to be carried out to fully understand this material.

The current state of knowledge from ARPES of materials such as TiSe_2_ [103], VSe_2_ [10,104], ReS_2_ [11,12,106], and ZrSe_2_ [93] may be regarded as being at a similar stage to the early studies of group VIB TMDCs. For example, the first ARPES on single layers WSe_2_ and MoS_2_ were performed by micro acquisition on exfoliated flakes [78,107,108]. After several studies and through growth optimization, WSe_2_ and MoS_2_ can now be grown epitaxially and advanced ARPES methods such as surface doping [9,93], spin-resolved ARPES [80] and time-resolved ARPES [98,99] can be carried out. These examples confirm that the union of mechanical transfer and spatially localized ARPES is crucial to the rapidly evolving 2D material world.

As reported above, by mechanical transfer it is possible to place samples on an arbitrary substrate and understand how their properties are modified by contact with other species. An example of this is the micro-ARPES study on suspended MoS_2_ compared to the more prevalent use of SiO_x_ which confirms the extreme sensitivity of 2D layers to the underlying substrate [109]. Examples of spatially-localized ARPES on CVD grown TMDCs has been reported for WS_2_, WSe_2_ and MoS_2_, where the band structure has been studied by micro-ARPES [78,81,110,111,112,113]. An interesting study of artificially built bi-layers of CVD-grown MoS_2_ has been carried out by micro-ARPES; the band structure changes as a function of the angle between the layers and a remarkable dependence of the angle with the electron effective mass and of the position of the bands has been found [112].

### 3.3. Two-Dimensional Heterostructures

Two-dimensional heterostructures are a vibrant research field with the principal aim of creating novel ultra-thin devices with unprecedented properties achieved by combining different 2D materials [3]. So far in this review, materials with different electronic structure have been discussed such as semi-metallic graphene, insulating *h*-BN and semiconducting TMDCs as well as phosphorene; these could be the components of future ultrathin devices utilizing exclusively 2D materials [14].

As reported above for the isolated 2D materials, some 2D heterostructures have also been synthesized by epitaxial methods, the advantage of this growth method is the possibility to perform complex ARPES analysis, but they are even more complex to synthesize than the isolated 2D materials. Several epitaxial routes are based on the use of graphene because it is so well-studied that advanced growth on top of it are possible, for example, TMDCs are deposited onto graphene by CVD or PVD. Epitaxial graphene on SiC represents an excellent example of graphene used as a basis for the growth of other 2D materials such as TMDCs: WSe_2_ [81], MoS_2_ [99], MoSe_2_ [114], and 2D chalcogenides such as single layer GaSe [115,116]. The latter material interacts greatly with the epitaxial graphene. ARPES has been used to estimate the graphene doping due to a charge transfer and a strong relation between GaSe band structure and the number of layers has been measured [115]. Epitaxial heterostructures that do not contain graphene are much rarer in the literature because the materials growth has not yet fully been disclosed and the deposition of different elements can cause undesired mixing and phase changes [64,117,118,119]. For example, the ability to grow and study GaSe heterostructures has been possible through the recent advances in its synthesis and enabled ARPES investigations on GaSe/GaAs [120] and GaSe/GaN [121]. The latter substrate has also been used as a substrate for MoS_2_ [122] and WSe_2_ [123] showing quite a strong interaction with the 2D layers. Interestingly, the reverse approach has also been studied with the deposition of GaN on top of TMDCs [124].

One way to overcome the disadvantage of complex CVD and PVD deposition methods to form heterostructures is the mechanical transfer methods, as mentioned above, which lends itself to spatially localized ARPES. For transferred layers, one of the most studied components for heterostructures is graphene or graphite. Indeed, graphenic flakes are now used as a conductive substrate [11,15] and as an ultrathin capping layer to preserve sensitive materials from oxidation [15].

The 2D heterostructures formed by mechanical exfoliation have been extensively used for the study of the electrical, magnetic and carrier transport properties of 2D materials [14,125,126,127,128]. Heterostructures composed of graphene and *h*-BN were the first to be studied, because it was clear that the properties of graphene were strongly influenced by the substrate. Hexagonal boron nitride being a flat, insulating material, with a dangling bond-free platform was a perfect candidate to study graphene pristine properties [125]. Spatially localized ARPES on graphene/h-BN verified the absence of graphene doping and also measured the replica of Dirac cones due to the Moiré superlattice on the graphene K points [129].

Graphene has been coupled with several TMDCs, a lot of studies have been focused on MoS_2_ [13,130,131,132] because it was one of the first materials in its class to be extensively studied, its fabrication is well known and is commercially available as large single crystals.

**Figure 8:**
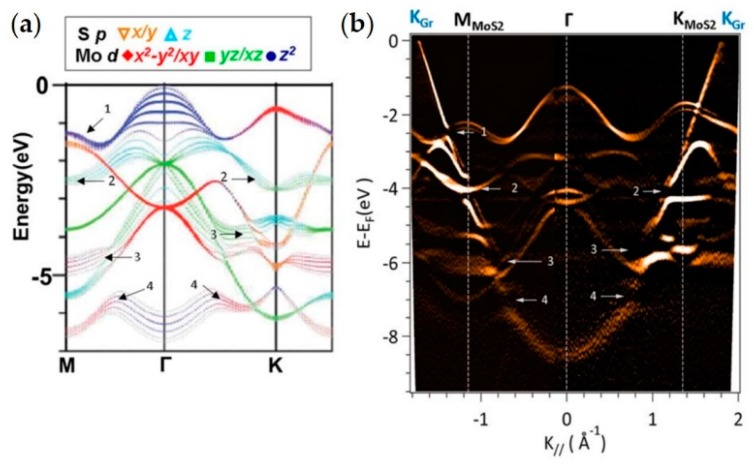
Example of ARPES from graphene and TMDC heterostructure with gap opening. (**a**) Calculated band structure of MoS_2_ with the orbital character of the individual bands color-coded. Adapted with permission from Ref. [133] Copyright 2012 American Physical Society.; (**b**) 2nd derivative of E-k ARPES spectrum of graphene/MoS_2_ bulk. The observed band gaps in the graphene π-band are labeled and their respective position with respect to the MoS_2_ band structure are indicated in (**a**). Reprinted with permission from Ref. [13] Copyright 2015 American Chemical Society.

The studies of heterostructures composed of graphene and MoS_2_ have been focused on studies of band offsets [130,131], mini-gap interactions [13,131] and alteration versus layer orientation [13,131,132]. A strong interaction between graphene and TMDC is visible in ARPES on the bands with an out-of-plane character. In Figure 8, the example of a polycrystalline azimuthally mis-oriented graphene transferred onto bulk MoS_2_ is reported. In this experiment the azimuthal disorder of transferred graphene made it possible to have on a single substrate several different alignments between the MoS_2_ and graphene. By using spatially-localized ARPES enabled the study of individual flakes and therefore made it possible to establish the band structure of the heterostructure as function of the layer orientation [13]. In Figure 8a the calculated band structure of MoS_2_ with the character of the bands for the Mo 5*d* and S 3*p* orbitals is presented. Figure 8b shows the nano-ARPES acquired from two flakes of graphene: for negative wavenumber graphene with Γ→K direction aligned with the Γ→M direction of MoS_2_, and for positive wavenumber graphene with Γ→K direction aligned with the direction Γ→K of MoS_2_. In the graphene π band there are clearly detectable gaps opening when crosses a MoS_2_ feature with an out-of-plane character. For example the gap “1” is due to Mo 5*d*_z2_ bands and it is visible only when the 2D materials are not aligned, while the gap “2” is visible in any of the two flakes reported in Figure 8b and it is due to S 3*p*_z_ orbitals [13]. Interestingly this phenomenon has not always been detected. For WSe_2_/graphite [15], MoSe_2_ and MoS_2_/graphene [13,114,131] it has been observed, however recently for WSe_2_/graphene it has not [81], so a more accurate investigation will be necessary to rule the effect of the heterostructures parameters, such as layer separation.

Few examples exist in the literature that report spatially localized ARPES on heterostructures that do not contain graphene, one recent example is the heterostructure WS_2_/*h*-BN [96]. This composite has been created by mechanical transfer and investigated by micro-ARPES. These measurements provide direct evidence of a trion quasiparticle and give access to both their energy and momentum dependence that is lacking from optical, tunneling or momentum-integrating transport measurements [96].

**Figure 9:**
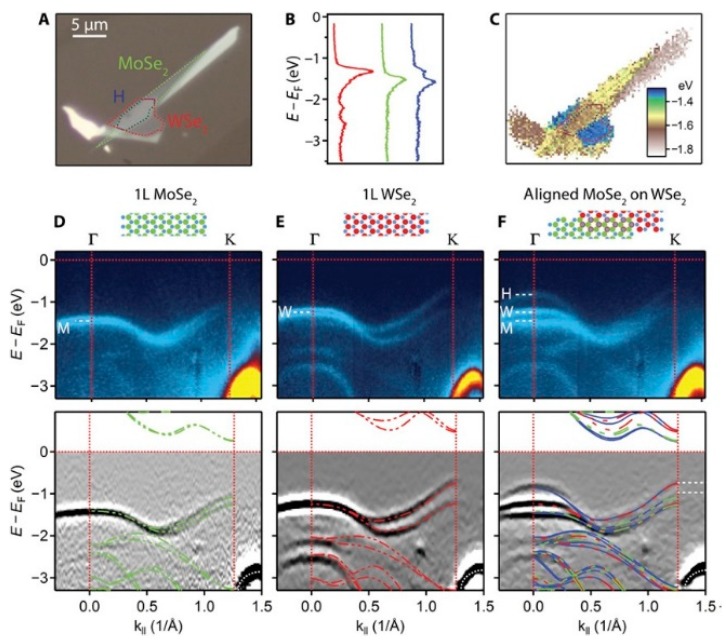
Example of all-TMDC heterostructure study by means of ARPES, PEEM, and optical spectroscopy. (**a**) Optical image showing mono-layer MoSe_2_ and WSe_2_ sheets, which overlap, with the MoSe_2_ on top, in an aligned hetero-bi-layer region (H). Their boundaries are indicated with color-coded dotted lines; (**b**) Angle-integrated spectra in each of the three regions; (**c**) Map of the energy of maximum emission; (**d**–**f**) Momentum slices along Γ-K in the three regions, (top) unprocessed and (bottom) twice-differentiated, with cartoons of the structures above. The superposed dashed colored lines are DFT calculations for the MoSe_2_ mono-layer (green), the WSe_2_ mono-layer (red), and the commensurate hetero-bi-layer (blue). The white dashes in the lower panel of (**f**) indicate the VBM in the MoSe_2_ and WSe_2_ mono-layers and hence the valence band offset. The white dashed lines in the upper panels of (**d**–**f**) mark the VBM in the isolated MoSe_2_ (M) and WSe_2_ (W) mono-layers and in the aligned hetero-bi-layer (H). Adapted from [15]. Reprinted with permission from AAAS.

The only example in the spatially-localized ARPES literature reported so far of all-TMDC heterostructures created by mechanical exfoliation has been reported in Ref. [15]; the main results are reproduced in Figure 9. In this work the heterostructure is formed from MoSe_2_/WSe_2_, both single layers, and has been studied by micro-ARPES. An optical microscope image of the sample is shown in Figure 9a; ARPES on the single 2D materials and on the heterostructure region is reported in Figure 9d–f. The single layer nature of the TMDC is confirmed by the VBM position at the K point for the isolated material. Spatially-localized ARPES acquisition on the heterostructure zone showed that in the proximity of the K point, the bands do not change position, while a new feature is formed at Γ. This is similar to what has been observed for a WSe_2_ bi-layer. The observations showing that the valence band edge remains at the K point and that the band alignment is type II are both extremely important for electronic and optoelectronic applications [15].

Complex heterostructures will become increasingly available for spatially localized ARPES studies because of the advances in the mechanical transfer technology used to form complex 2D devices for optical, carrier transport, magnetic, and spin property characterization [3,14,134,135,136,137]. It is expected that band structure measurements will integrate information derived from other microscopic techniques and vice-versa. For example, optical spectroscopies can provide information on the number of layers, band positions, exciton formation and the type of band alignment [15,64,138,139,140,141,142,143], but only ARPES allows the direct visualization of the band structure and can elucidate the carrier dynamics [15,96,97].

## 4. Conclusions

In this review, the principles of spatially-localized ARPES have been reported. Experimental measurement configurations, from early ARPES systems to the most advanced state-of-the-art instruments for micro and nano-ARPES have been presented. The technical challenges of using these tools have been discussed and both synchrotron and laboratory-based instruments have been introduced.

ARPES using laboratory-based equipment is feasible with crystal domains a few microns across, the light intensity available is typically lower than those at synchrotron facilities, but long acquisitions are possible because longer measurement times can be made. Also, they are very stable instruments that are not affected by beam oscillation or downtime. Laboratory-based instruments can also be extremely useful for evaluating new sample materials and for performing preliminary analyses prior to accessing synchrotron beamtime. Spatially localized ARPES synchrotron beamlines allow the exploration of the band dispersion in k_z_, which is particularly useful for bulk crystals [12,85], and for polarization-sensitive investigations [64]. Furthermore, the application of ARPES to nano-sized regions is achievable with extremely fine focusing of the beam, especially at facilities that do not require any sample rotation due to the use of state-of-the-art hemispherical analyzers.

In this context the reported examples in Figure 4, Figure 6, and Figure 7 exemplify the importance of sampling entire sectors of a band structure and not only in a few high symmetry directions. The ability to acquire a major sector of the Brillouin zone makes it possible to observe isoenergetic surfaces, such as the Fermi surfaces shown in Figure 6 [10] and the VBM in Figure 7 [11], and to extract energy versus k_//_ profiles in arbitrary directions as is done in Figure 4 for the vHS [7].

Examples of graphene, TMDCs and 2D heterostructures experiments have been used to showcase the potential of spatially-localized ARPES for 2D material investigations. Because spatially-localized ARPES is directly linked to the lateral dimensions of the material, the preparation methods for ultra-thin layers has been discussed, highlighting how mechanical exfoliation and transferred films represent the most promising methods for micro-ARPES characterizations.

Graphene has been discussed in detail because it is the prototype 2D layer and the model material to which spatially localized ARPES has been applied. ARPES on graphene allows one to visualize directly the Dirac cones, which are characteristics of its semi-metallic structure, probing its doping level [7], interaction with surrounding ultra-thin layers, Fermi velocity [30], and many-body effects [31]. These works opened the way to similar acquisition being performed on a variety of different 2D materials.

TMDCs have been studied for their novel optical, electron carrier transport and spin properties. Spatially-localized ARPES has been used to observe the changes in the band structure as function of the number of layers, as illustrated in Figure 5 [9], to observe complex phenomena such as CDWs (Figure 6) [10], and also reveal unusual symmetries (Figure 7) [11]. Nowadays, TMDCs as isolated materials and as components of heterostructures are probably the most studied 2D materials. Advanced ARPES studies, such as spin-resolved and time-resolved acquisitions, have been undertaken on large samples of TMDCs. It is foreseeable that the future evolution of spatially-resolved ARPES will allow spin- and time-resolved acquisitions to be made on much smaller samples.

The field of 2D heterostructures is probably the most interesting subject for spatially-localized ARPES. In this review are reported several heterostructures based on graphene which itself can be used as a conductive substrate [11,15], a protective capping layer [15] or even a contact for future ultra-thin devices [14]. Its ability to provide protection from oxidation for air sensitive samples may be the only feasible way to measure some technologically promising 2D materials such as phosphorene [75].

To date, the analyses by spatially-localized ARPES have been used for only a few kinds of 2D heterostructures, and rarely have been made solely from TMDCs [15]. However, in the future, it is expected that this spectromicroscopy technique will be used regularly in conjunction with a wide range of complimentary optical, electrical and magnetic techniques.

The possibility to observe the band structure in a confined space created by the overlapping of microscopic 2D sheets will allow the study of a whole range of phenomena from band alignments to many-body effects and soon also spin polarization and carrier dynamics. The prospects are very good for the spatially resolved ARPES technique to revolutionize the way in which promising 2D materials and heterostructures are studied and analyzed.

## Figures and Tables

**Table 1:** Different type of ARPES: advantages, disadvantages and future upgrades.

Type of ARPES		Advantages	Disadvantages	Future Upgrades
Conventional		Easy operation in laboratory and synchrotron facility. Best energy resolution for ARPES.	Requires large samples and several sample rotations.	Electron analyzers with deflectors can scan a sector of k-space without moving the sample.
Spatially-resolved	Synchrotron beamlines	Strong light intensity, possibility to perform ARPES over tens of nanometers, photon versatility, *k_z_* sampling.	Requires: precise sample movements, rotations, access to specialized synchrotron beamlines.	Electron analyzers with deflectors can scan a sector of k-space without moving the sample.
	EF-PEEM	Full-wavevector ARPES with no sample rotation or movement involved. These can be located in stand-alone laboratories with excellent stability.	Limited photon tunability, no *k_z_* sampling, lower signal intensity and larger sampling areas with respect to synchrotron nano-ARPES beamlines.	Time-resolved measurements by TOF PEEM, spin-resolved measurements with state-of-the-art detectors.
Spin-resolved		ARPES with spin-resolution.	Intrinsic low signal, limited to large crystal or epitaxial films.	Integration of spin-sensitive detectors in EF-PEEM to perform full-wavevector ARPES and spatially-resolved measurements.
Time-resolved		Studies of: charge carrier dynamics, band gap, empty states, time-dependent phenomena.	Low signal, limited to large crystal or epitaxial films. Complex lasers or time-resolved setups required as excitation sources. Space charge effects.	Advances in TOF PEEM setups and time-resolved light sources to perform spatial- and time-resolved studies.
